# Multiple domestication events explain the origin of *Gossypium hirsutum* landraces in Mexico

**DOI:** 10.1002/ece3.9838

**Published:** 2023-03-08

**Authors:** Melania Vega, Christian Quintero‐Corrales, Alicia Mastretta‐Yanes, Alejandro Casas, Victorina López‐Hilario, Ana Wegier

**Affiliations:** ^1^ Genética de la Conservación, Jardín Botánico Instituto de Biología, Universidad Nacional Autónoma de México Ciudad de México Mexico; ^2^ Posgrado en Ciencias Biológicas Universidad Nacional Autónoma de México Ciudad de México Mexico; ^3^ Departamento de Botánica Instituto de Biología, Universidad Nacional Autónoma de México Ciudad de México Mexico; ^4^ Comisión Nacional para el Conocimiento y Uso de la Biodiversidad (CONABIO) Ciudad de México Mexico; ^5^ Consejo Nacional de Ciencia y Tecnología (CONACYT) Programa de Investigadores e Investigadoras por México Ciudad de México Mexico; ^6^ Instituto de Investigaciones en Ecosistemas y Sustentabilidad Universidad Nacional Autónoma de México Morelia Mexico; ^7^ Tejedoras Amuzgas De Piedra Pesada Guerrero Mexico

**Keywords:** chloroplast evolution, conservation genetics, cotton, crop wild relatives, domestication

## Abstract

Several Mesoamerican crops constitute wild‐to‐domesticated complexes generated by multiple initial domestication events, and continuous gene flow among crop populations and between these populations and their wild relatives. It has been suggested that the domestication of cotton (*Gossypium hirsutum*) started in the northwest of the Yucatán Peninsula, from where it spread to other regions inside and outside of Mexico. We tested this hypothesis by assembling chloroplast genomes of 23 wild, landraces, and breeding lines (transgene‐introgressed and conventional). The phylogenetic analysis showed that the evolutionary history of cotton in Mexico involves multiple events of introgression and genetic divergence. From this, we conclude that Mexican landraces arose from multiple wild populations. Our results also revealed that their structural and functional chloroplast organizations had been preserved. However, genetic diversity decreases as a consequence of domestication, mainly in transgene‐introgressed (TI) individuals (π = 0.00020, 0.00001, 0.00016, 0, and 0, of wild, TI‐wild, landraces, TI‐landraces, and breeding lines, respectively). We identified homologous regions that differentiate wild from domesticated plants and indicate a relationship among the samples. A decrease in genetic diversity associated with transgene introgression in cotton was identified for the first time, and our outcomes are therefore relevant to both biosecurity and agrobiodiversity conservation.

## Graphical abstract

Domestication of cotton landraces in Mexico. Changes in chloroplast genetic diversity related to cotton domestication and management.

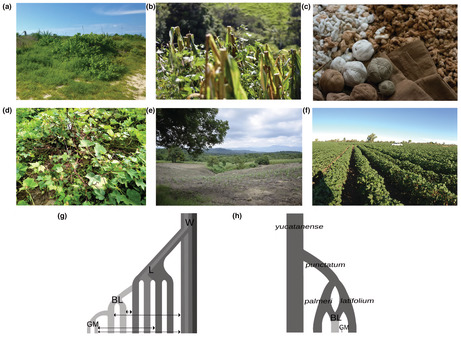

## 1 INTRODUCTION

**FIGURE 1:**
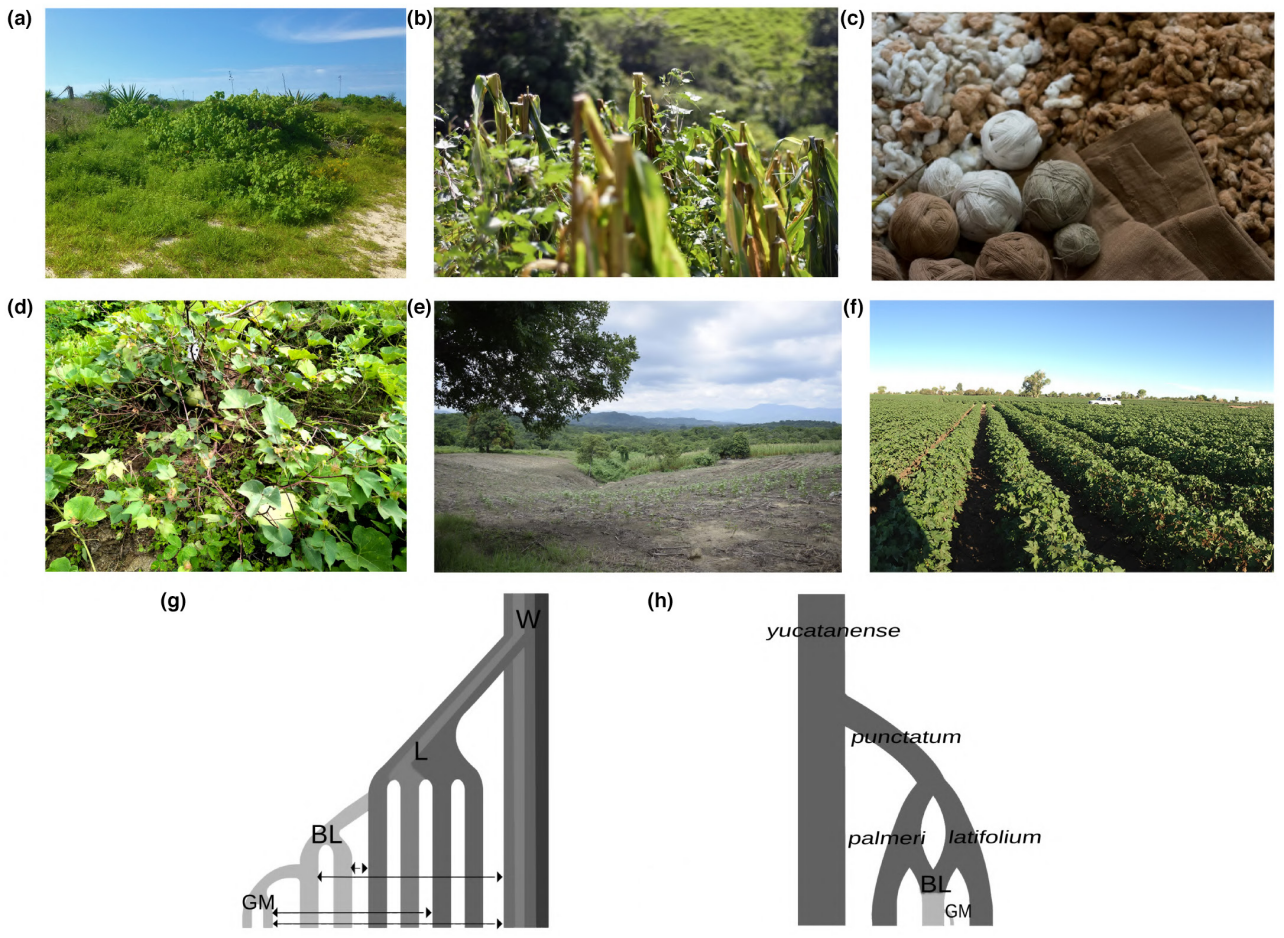
Condition in which the wild‐to‐domesticated cotton complex is developed in Mexico. (a) Wild population from the Yucatán Peninsula. (b) The white variety of cotton inside a corn crop in Oaxaca, Mexico. (c) Fiber color and white, brown, and green threads. (d) The brown variety of cotton among a pumpkin crop in Oaxaca, Mex. (e) The white variety of cotton in monoculture when the land maintains a slope. (f) Monoculture of transgenic cotton in Mexicali, Baja California, Mex. (g) Hypothesis that suggests domestication processes in which the breeding lines (BL) developed from local races (L), these in turn, arose from wild metapopulations (W). BL, L and actual genetically modified varieties (GM) have maintained continuous gene flow and introgression with their wild relatives (indicated by arrows). (h) Hypothesis proposed by Brubaker and Wendel (1994) in which the north‐western Yucatán Peninsula is suggested as the primary site for the earliest stages of domestication. *Yucatanese* corresponds to a wild race of cotton recognized as *truly wild*, from which *punctatum*, *palmeri*, and *latifolium* emerged, which are recognized as wild or agronomically primitive forms, from which BL were subsequently obtained.

Several studies have documented multiple independent domestication events and a recurrent gene flow among crop populations and their wild relatives. These events have often occurred in Mesoamerica, a region recognized as the main center of origin of crop domestication, mainly induced by the diffusion of seeds and vegetative propagules carried out by humans (Kraft et al., 2014; Moreno‐Letelier et al., 2020; Roullier et al., 2013; Zizumbo‐Villarreal & Colunga‐GarcíaMarín, 2010). The cultural and biological aspects related to the use and management of plants have caused the formation of wild‐to‐domesticated complexes, which are formed by wild populations; landraces; breeding lines (BL); feral individuals; transgene‐introgressed (TI) wild populations; TI‐landraces; and genetically modified BL (Alavez et al., 2021; Velázquez‐López et al., 2018; Figure 1). Wild‐to‐domesticated complexes have been documented in beans, maize, tomato, pumpkin, and avocado crops, in which genetic diversity is differently maintained and modified by humans while also being influenced by natural evolutionary processes (Castellanos‐Morales et al., 2019; Chen et al., 2009; Moreno‐Letelier et al., 2020; Motta‐Aldana et al., 2010; Razifard et al., 2020). Within the wild‐to‐domesticated complexes, local varieties have main roles in both processes of domestication and conservation of genetic resources. The introgression of alleles from landraces to BL is often more successful than the introgression from wild populations, since the former are better adapted to agricultural conditions and have less fertility or sterility issues (Prohens et al., 2017). Local varieties usually have more phenotypic and genetic diversity than BL as well as higher levels of gene flow with wild relatives and BL, promoted through seed exchange and/or breeding (Camacho‐Villa et al., 2005; Casañas et al., 2017; Taitano et al., 2019).

*Gossypium hirsutum* L. (1763) is a cotton species with high biological, economical, and cultural importance worldwide. Mexico has been recognized as its center of origin, due to the occurrence of wild populations along its coastal dunes (Pérez‐Mendoza et al., 2016; Ulloa et al., 2005; Wegier et al., 2011). Besides, Mexico has been considered the most ancient domestication area of this cotton species. Archeological evidence suggests that cotton has been used for more than 4000 years in Mesoamerica, and a high phenotypic and genotypic variation has been reported in local cotton varieties (Smith & Stephens, 1971). Historical records such as codices and chronicles of the Spanish Conquest indicate that cotton was already cultivated and used throughout the current Mexican territory by pre‐Hispanic cultures, with purposes such as weaving textiles and practicing rituals (FAMSI, 2021a, 2021b; Ruiz y Sandoval, 1884; Sahagún, 1829). In the 19th and 20th centuries, intensive cotton cultivation programs were promoted in Mexico. During this period, cotton was considered the “white gold,” due to its high economic relevance (Aboites, 2013). However, the intensive production of cotton crops faced significant losses caused by pests. Genetically modified cotton began to be cultivated in 1996, looking for pest problems solutions (James, 2016). Currently, the production of transgenic cotton has replaced the farming of conventional BL, but numerous indigenous and local communities continue cultivating native landraces within the maize polyculture system called *milpa*, as well as in monoculture systems.

The *milpa* is a traditional agricultural system, as well as a culturally important socio‐ecological system in Mexico. Plant species cultivated in the milpa are selected to favor their growth among themselves and agricultural management is aimed at maintaining healthy soil (Moreno‐Calles et al., 2013). Maize, beans, and squash are the most frequently cultivated crops in the *milpa*, but in Mexican territory, it is possible to find other species intercropped (Moreno‐Calles et al., 2013; Novotny et al., 2021). In some *milpas*, cotton landraces are associated with corn or squash (Figure 1). Local varieties of cotton include brown (or *coyuchi*), green, and white cotton, which remain important to the cultural and economic activities of indigenous peoples and local communities (Pérez‐Mendoza et al., 2012).

Research regarding cotton domestication, history, and production in Mexico is extensive (Aboites, 2013; Mikulska, 2001; Ruiz y Sandoval, 1884; Sahagún, 1829). However, it is necessary to integrate information about ecological and evolutionary processes and threats to achieve cotton in situ conservation. Although wild populations and domesticated plants have different evolutionary histories, they can maintain gene flow and show introgression, since their reproductive system is compatible (Brubaker & Wendel, 1994; Velázquez‐López et al., 2018). Therefore, as transgenic varieties are sown in northern Mexico, the gene flow of domesticated alleles and transgenes became a worrying issue (Wegier et al., 2011). The Cartagena Protocol on Biosafety of the Convention on Biological Diversity indicates that each country is responsible for regulating permits for the management and release of genetically modified organisms (GMOs). Also, for taking the necessary measures to avoid negative effects on health or biological diversity due to the use of GMOs, prioritizing the protection of species in their center of origin and genetic diversity (CBD, 2003). Mexican laws and regulations have been implemented to promote the protection of cotton genetic resources. These are the cases of the norms of the *Ley de bioseguridad de organismos genéticamente modificados* (Diario Oficial de la Federación, 2005). Proposals of strategies for the conservation of cotton genetic resources and scientific information, especially that providing genetic and evolutionary data, are complementary to the laws (Tobón‐Niedfeldt et al., 2022).

Wild populations and landraces are essential genetic reservoirs to the continued processes of domestication and new goals of breeding of current crops (Tobón‐Niedfeldt et al., 2022). Recent assessments of the extinction risk of crop wild relatives indicate that wild populations of *G. hirsutum* are vulnerable to extinction, according to IUCN criteria (Goettsch et al., 2021; Wegier et al., 2019). These populations are considered under special protection by the Mexican law NOM‐059‐SEMARNAT (Diario Oficial de la Federación, 2019). This protection category includes species or populations threatened and whose recovery and conservation need to be promoted. The main threat endangering wild populations is gene flow, and the introgression of domesticated alleles and transgenes from crops (Wegier et al., 2019). Transgenic varieties of cotton began to be planted in Mexico in 1996, and Wegier et al. (2011) evidenced recent gene flow from crops to wild populations by identifying the presence of the Cry1ab/Ac, Cry2A, CP4EPSPS, and PAT/bar proteins through immunoassays. The introgression of transgenes into wild populations was documented by Vázquez‐Barrios et al. (2021) who demonstrated that it is possible to observe the presence of transgenes after 10 years from their first report in wild populations. These authors also found that the transgene frequencies have changed and are affecting ecological interactions.

Gene flow and introgression of domesticated alleles and transgenes into wild populations can induce genetic erosion, changes in the populations' structure, phenotypic modifications, and alterations in survival rates (Jin et al., 2018; Lu, 2013). Phenotypic modifications and changes in survival rates were observed experimentally by breeding wild pumpkin (*Cucurbita argyrosperma* ssp. *sororia*) with transgenic pumpkin varieties (*Cucurbita pepo* Virus Resistant Transgenic). The result showed that germination and survival rates were significantly higher in the parental lines than in hybrids, while the germination time was higher and more variable in hybrids compared with parental lines (Cruz‐Reyes et al., 2015).

The present study is the first report about the presence of transgenes in cotton landraces. The loss or modification of genetic diversity in landraces due to transgene introgression has been scarcely studied. However, concern about genetic erosion associated with the use of modern or transgenic cultivars has been raised indirectly, since the use of modern cultivars and the tendency to homogenize diversity in monocultures has caused disuse and reduction in the number of landraces (Guzzon et al., 2021). Another example is bean landraces in Campeche, Mexico, one of the centers of genetic diversity of this crop species. There, Martínez‐Castillo et al. (2012) observed that the reduction of local varieties caused a reduction in allelic diversity from 1979 to 2007 (diversity of Nei *H* = 0.18 and 0.05, respectively). Because Mexico is the center of origin, domestication, and diversity of cotton, we can raise the problem of cotton in this country as an important issue concerning biosafety. The possible loss or modification of diversity related to the presence of transgenes, coupled with the antecedents observed in bean landraces, motivated us to include landraces within the priority genetic resources that must be conserved similarly to wild populations.

Chloroplast DNA sequences have been some of the most widely used molecular markers when studying domesticated plants. Their conservative nonrecombinant nature allows reconstructing phylogenetic relationships among species while maintaining the diversity needed to distinguish different populations (Cheng et al., 2019; Dong et al., 2012; Nock et al., 2019; Tamburino et al., 2020; Yin et al., 2021). Previous studies of the structure of cotton's chloroplast genome have helped to explain the phylogeographic relationships of the *Gossypium* species and their evolutionary processes (Chen et al., 2017; Cheng et al., 2020; Wu et al., 2018; Xu et al., 2012). Genus *Gossypium* is divided into eight diploid genome groups that are named with the letters A–G, and K, and one allopolyploid clade that refers to AD genome type (Wang, Wendel, & Jinping, 2018). These genomic groups have been defined through taxonomic and phylogenetic analysis (Wang, Wendel, & Jinping, 2018), although deciphering which parental species was the donor of the chloroplast possessed by species of the AD genotype has been a complex task. For this reason, it has been very useful to study the complete chloroplast genome (Li et al., 2014). Studies with the whole chloroplast genome of cotton confirmed that genome A is the chloroplast donor among parental species (Li et al., 2014). Furthermore, intraspecific genetic analyses in Mexican populations have shown that the genetic diversity of wild varieties is greater than that of domesticated populations (Wegier et al., 2011). Artificial selection and bottlenecks associated with domestication processes have commonly narrowed the genetic variation of crops (Meyer & Purugganan, 2013; Smýkal et al., 2018). This observation is important for understanding the evolution of the wild‐to‐domesticated cotton complex in Mexico. Despite the importance of the chloroplast genome and its functions for plants to develop correctly, the study of diversity and structural changes in relation to the insertion of transgenes has been scarcely studied because *Agrobacterium‐*mediated transformations in cotton are designed to modify the nuclear genome. However, due to the interaction between cytoplasmic and nuclear genomes, we cannot rule out the possible effects of transgene introgression on the chloroplast (Zhao et al., 2019). For instance, (Stegemann et al., 2003) conducted experimental crosses between transgenic tobacco varieties with chloroplast modifications (inserting the *ntpII* and *aad* genes) showing that, eventually, the transgenes are transferred to the nuclear genome, where the regulatory machinery is more effective. This study, proved that DNA escapes from the chloroplast and that its integration into the nuclear genome occurs more frequently than generally considered, therefore providing a mechanism that not only causes intraspecific but also intraorganism genetic variation (Stegemann et al., 2003).

Given that local varieties represent an important part of the genetic diversity of the wild‐to‐domesticated complex, they complement the current view on the evolutionary history of cotton in Mexico. Without considering the study of landraces and with a limited knowledge regarding the wild populations spread throughout the Pacific coast, it has been suggested that cotton domestication originated in the Yucatán peninsula and later spread to the rest of Mexico and other countries (Brubaker & Wendel, 1994; Grover et al., 2020; Yuan et al., 2021). Based on the analysis of the divergence between wild and domesticated populations, we examined whether cotton domestication occurred in a single or in multiple events (Figure 1g,h). Furthermore, we explored the structure and diversity of the chloroplast genome, in order to expand the understanding of the genomic effects of the use and management processes of cotton. This study aspires to provide useful information for understanding the process of domestication of cotton and bases for the conservation of valuable genetic resources.

## 2 MATERIALS AND METHODS

### 2.1 Sampling and data acquisition

Wild and landrace samples of *G. hirsutum* were collected from Nayarit, Guerrero, Oaxaca, and Yucatán during 2005, 2015, and 2019 (Table 1). Genomic DNA from 16 samples was extracted from young leaves and seeds using the CTAB protocol (Wegier et al., 2011). Sequencing libraries were constructed using the TruSeq PCR Free (350) kit and sequenced on the NovaSeq 6000 Illumina platform with paired‐end reads with a 150 bp length. Library construction and sequencing were carried out at the Macrogen laboratory, USA. Additional raw reads of wild and breeding lines samples were acquired from NCBI: *G. hirsutum yucatanense* TX‐02094 (SRR1975549), *G. hirsutum* Guatemala TX‐0231 (SRR1536369), *G. hirsutum* TM‐1 (SRR1534688), *G. hirsutum* Stoneville474 (SRR1536367), *G. hirsutum* Coker‐312 (SRR1536365), *G. hirsutum* Fibermax832 (SRR1536364), and *G. hirsutum* Acala Maxxa (SRR617482).

**TABLE 1:** Features of the genomes of chloroplast extracted from samples sequenced of wild, landraces, and breeding lines cotton (*Gossypium hirsutum*).

Sample	Domestication status	Size (base pair)				Accession ID
		Total	LSC	SSC	IR	
W_yucatan1^a^	Wild	160,281	88,778	20,298	25,601	MZ391798
**W_yucatan2** ^a^	Wild	160,282	88,779	20,298	25,601	MZ391799
**W_yucatan3** ^a^	Wild	160,294	88,791	20,298	25,601	MZ391800
**W_yucatan4** ^a^	Wild	160,294	88,791	20,298	25,601	MZ391801
**W_yucatan5** ^a^	Wild	160,288	88,785	20,298	25,601	MZ391802
W_yucatanense^b^	Wild	160,270	88,767	20,298	25,601	BK059185
W_banderasbay^a^	Wild	160,305	88,815	20,285	25,601	MZ391803
W_guatemala^b^	Wild	160,280	88,777	20,298	25,601	BK059346
L_oaxbrown1^a^	Landrace	160,307	88,817	20,298	25,601	MZ391804
**L_oaxbrown2** ^a^	Landrace	160,296	88,812	20,279	25,601	MZ391805
**L_oaxbrown3** ^a^	Landrace	160,296	88,812	20,279	25,601	MZ391806
**L_oaxbrown4** ^a^	Landrace	160,296	88,812	20,279	25,601	MZ391807
**L_oaxbrown5** ^a^	Landrace	160,296	88,812	20,279	25,601	MZ391808
L_oaxgreen^a^	Landrace	160,278	88,781	20,290	25,602	MZ391809
**L_oaxwhite** ^a^	Landrace	160,295	88,812	20,278	25,601	MZ391811
L_guebrown^a^	Landrace	160,273	88,776	20,290	25,602	MZ391810
L_guegreen^a^	Landrace	160,278	88,781	20,290	25,602	MZ391812
L_guewhite^a^	Landrace	160,278	88,781	20,290	25,602	MZ391813
BL_tm1^b^	Breeding line	160,294	88,811	20,278	25,601	BK059347
BL_stoneville474^b^	Breeding line	160,296	88,812	20,279	25,601	BK059348
BL_Coker312^b^	Breeding line	160,295	88,811	20,279	25,601	BK059349
BL_fibermax832^b^	Breeding line	160,296	88,812	20,279	25,601	BK059350
BL_acalamaxxa^b^	Breeding line	160,295	88,811	20,279	25,601	BK059351

*Note*: The length of all samples agrees with the reference genome of species (160,301 bp) with some variations related to changes in LSC and SSC regions. Samples highlighted in bold indicate transgene‐introgressed individuals.

Abbreviations: IR, inverted repeat; LSC, large single copy; SSC, short single copy.

^a^ Plastomes extracted from samples sequencing in this study.

^b^ Plastomes extracted from sequenced samples available in NCBI.

### 2.2 Detection of transgenes in the samples

To characterize the presence of transgenic samples among the collection, we performed a Polymerase Chain Reaction (PCR) assay. Transgenic events released in Mexico, related to constructions of lepidopteran resistance and one of herbicide tolerance (*Cry1Ab/Ac*, *Cry2Ab*, and *CP4EPSPS*), were verified using the following primers from Eurofins Scientific:

(1) *Cry1Ab/Ac* (F 5′ ACCGGTTACACTCCCATCGA 3′, R 5′ CAGCACCTGGCACGAACT 3′).

(2) *Cry2Ab* (F 5′ CAGCGGCGCCAACTCTACG 3′, R 5′ TGAACGGCGATGCACCAATGTC 3′).

(3) *CP4EPSPS* (F 5′ GCATGCTTCACGGTGCAA 3′, R 5′ TGAAGGACCGGTGGGAGAT 3′).

Furthermore, we searched for transgenes in all 23 samples with blastn task from BLAST+ V 2.9.0–2 (Camacho et al., 2009) using a reference database with the specific sequences detected in Mexican cotton (Hernández‐Terán et al., 2019).

### 2.3 Chloroplast genome assembly, structure, and genetic diversity analysis

Sequencing raw data results and raw data acquired from NCBI made up a total of 954 GB of information. Each sample had 10–50× sequencing coverage and the quality of the reads was examined using FastQC v.0.11.7 (Andrews et al., 2010). We used pipeline GetOrganelle V 1.7.3.4 (Jin et al., 2020) to extract and assemble the chloroplast genome. The program performs a de novo assembly using seed and genome sequences as references. Here, we used the Ribulose 1,5‐bisphosphate (RuBP) sequence from *Zea mays* and *Gossypium hirsutum* Coker 310 FR complete chloroplast genome (GenBank: NC_007944; Lee et al., 2006), as seed and reference sequences, respectively. The annotation of each genome was performed using GeSeq (Tillich et al., 2017) and CPGAVAS2 (Shi et al., 2019), using the *Gossypium hirsutum* Coker 310 genome as references. OrganellarGenomeDRAW (Greiner et al., 2019) was used to plot the chloroplast structure for each assembly and to compare the LSC‐IR‐SSC‐IR region boundaries. The genomic rearrangements and homologous regions were detected with the alignment of the chloroplast using Mauve algorithm in Mauve V 2.4.0 (Darling et al., 2010) with default parameters.

To identify single nucleotide variants (SNVs), Indels, and short tandem repeats (STRs) we aligned the raw reads with the reference genome of *G. hirsutum* cocker 310 FR using Burrows‐Wheeler Aligner 0.7.17‐r1188 (Li & Durbin, 2009). We used Samtools 1.10 (Li et al., 2009), Picard 2.6.2 (http://broadinstitute.github.io/picard/), and NGSEP 3.3.0 (Tello et al., 2019) as format converters, for sorting files and to identify genomic variants. The genomic variability was compared between samples and between the sum of genomic variants in wild, TI‐wild, landrace, TI‐landrace, and BL genomes. Nucleotide diversity (π), N_ST_ index, and the Tajima's D test were calculated using DnaSPv6 (Rozas et al., 2017); and synonym and nonsynonym substitution rates (dN/dS) were calculated with PAML through PAL2NAL (Suyama et al., 2006; Xu & Yang, 2013). The genetic diversity and selection were compared between the groups with different degrees of management, and between these and the introgressed groups. We examined population structure by performing a Bayesian spatial analysis using the package RhierBAPS (Cheng et al., 2013; Tonkin‐Hill et al., 2018). The haplotype diversity was calculated using DnaSPv6 (Rozas et al., 2017). In addition, we analyzed the evolutionary history and relationships among the haplotypes and gene flow by constructing a minimum‐spanning network of haplotypes using TCS in the software POPART (Leigh & Bryant, 2015).

### 2.4 Phylogenetic analyses and divergence time estimation

For the phylogenetic inferences, we downloaded *Gossypium's* chloroplast genomes from NCBI (Table S1) and used the *Theobroma cacao* sequence as the outgroup. All sequences were aligned using MAFFT V 7.0 (Katoh et al., 2019) and edited in MEGA‐X V 10.2.4 (Kumar et al., 2018). In order to choose the best evolutionary model for our data, we used JModeltest (Darriba et al., 2012) and the corrected Akaike Information Criterion (AICc). Three different datasets were prepared for phylogenetic analyses: complete chloroplast genome (hereafter “full chloroplast” dataset); SSC chloroplast fragment (“SSC” dataset), and LSC + IRs concatenate fragments (“LSC + IR” dataset, concatenation was performed in MEGA‐X). For each dataset, maximum‐likelihood analyses were filed using RaxML with the General Time Reversible (GTR) model plus Gamma and 1000 bootstraps in CIPRES Science Gateway V 3.3 (Miller et al., 2010). Tree visualization was performed using FigTree V1.4.3 (http://tree.bio.ed.ac.uk/software/figtree/). Furthermore, we developed a phylogenetic network using SplitsTree V 5.0.20 with 1000 bootstraps and samples of the wild‐to‐domesticated complex; we also included representative samples of each genetic group described as a kind of *Gossypium* as an outgroup.

In order to calculate the divergence time, we took into consideration all the coding gene sequences of the assembled plastomes present in this study, and we included *G. herbaceum*, *G. logicalyx*, *G. stockii*, and *G. thurberi* as external groups. We performed two independent runs of 400,000 chain length sampling of each 1000 chains. We used the gamma site with the GTR evolutionary model and estimated the nucleotide substitution rate using JModeltest (Darriba et al., 2012). We chose a relaxed clock log‐normal model together with the calibrated yule model. Three calibration points were set according to the cotton chloroplast genome type divergence: F‐clade (7.23 mya), E‐clade (4.28 mya), and A + AB‐clade (1.53 mya) according to Chen et al's research (2016). We also used BEAUti from BEAST 2.0 (Bouckaert et al., 2019; Suchard et al., 2018) to build the xml file and performed a Convergence chain test with Tracer 1.7 (Rambaut et al., 2018). The log and tree files from both runs were concatenated using Logcombiner and the best tree was extracted with Treeannotator with 15% burnin trees, maximum clade credibility tree, and mean heights. The target tree was plotted in R (Heibl, 2008) with the phyloch and strap V 1.4 packages (Bell & Lloyd, 2014).

## 3 RESULTS

### 3.1 Selection signature and genetic diversity of wild, landrace, and breeding lines plastomes

Through the PCR tests and results obtained by reviewing the sequences with the BLAST tools, it was confirmed whether or not the samples contained transgenes. The samples with evidence of introgression are: W_yucatan2‐5 with *CP4EPSPS* transgene; L_oaxbrown2‐5 and L_oaxwhite with *CP4EPSPS*, *Cry1Ab/Ac*, *Cry2Ab* transgenes. The sequences found in each sample are shown in Table 2.

**TABLE 2:** Sequences of the transgenes identified using the BLAST tools.

Sample	Transgenes sequences
W_yucatan2	*CP4EPSPS*: AGGACCGAGTTGGAGATGATCAGCTTGGCTGTTCACCGTAATCTGCTTCGTTTGATTGGTTATTGTGCTACTTCTAACGAAAGACTCCTTGTCTACCCTTACATGTCTAATGGCAGTGTTGCATCCAGGCTTAGAGGTTTGCTCAAAGAC
W_yucatan3	*CP4EPSPS*: AACTTGTTTGGGTCTTTGAGCAAACCTCTAAGCCTGGATGCAACACTGCCATTAGACATGTAAGGGTAGACAAGGAGTCTTTCGTTAGAAGTAGCACAATAACCAATCAAACGAAGCAGATTACGGTGAACAGCCAAGCTGATCATCTCC
W_yucatan4	*CP4EPSPS*: TCATTTACAGTAAAAAATTCCATTCCCAAATTCAAAAAAGAACTTGTTTGGGTCTTTGAGCAAACCTCTAAGCCTGGATGCAACACTGCCATTAGACATGTAAGGGTAGACAAGGAGTCTTTCGTTAGAAGTAGCACAATAACCAATCAAA
W_yucatan5	*CP4EPSPS*: AAGAACTTGTTTGGGTCTTTGAGCAAACCTCTAAGCCTGGATGCAACACTGCCATTAGACATGTAAGGGTAGACAAGGAGTCTTTCGTTAGAAGTAGCACAATAACCAATCAAACGAAGCAGATTACGGTGAACAGCCAAGCTGATCATCT
L_oaxbrown2	*Cry1Ab/Ac*: GATCGAGAACAACACCGACGAGCTTAAGTTCTCCAACTGCGTCGAGGAAGAAATCTATCCCAACAACACCGTTACTTGCAACGACTACACTGTGAATCAGGAAGAGTACGGAGGTGCCTACACTAGCCGTAACAGA
L_oaxbrown3	*Cry2Ab*: TCTTCAATCCCCACGACGACGAAATCGGATAAGCTCGTGGATGCTGCTGCGTCTTCAGAGAAACCGATAAGGGAGATTTGCGTTGACTGGATTTCGAGAGATTGGAGATAAGAGATGGGTTCTGCACACCATTGCA
L_oaxbrown4	*Cry1Ab/Ac*: AGGCATAGTCAGCAGGAACGGAAGGAGCTTCGTTGTAACCTCTGTTACGGCTAGTGTAGGCACCTCCGTACTCTTCCTGATTCACAGTGTAGTCGTTGCAAGTAACGGTGTTGTTGGGATAGATTTCTTCCTCGAC *Cry2Ab*: ATGGTGTGCAGAACCCATCTCTTATCTCCAATCTCTCGAAATCCAGTCAACGCAAATCTCCCTTATCGGTTTCTCTGAAGACGCAGCAGCATCCACGAGCTTATCCGATTTCGTCGTCGTGGGGATTGAAGAAGAG *CP4EPSPS*: AAACATGAAGGACCTGTGGGAGATAGACTTGTCACCTGGAATACGGACGGTTCCAGAAAGACCAGAGGACTTACGAGCAGT
L_oaxbrown5	*Cry2Ab*: CGACGACGAAATCGGATAAGCTCGTGGATGCTGCTGCGTCTTCAGAGAAACCGATAAGGGAGATTTGCGTTGACTGGATTTCGAGAGATTGGAGATAAGAGATGGGTTCTGCACACCATTGCAGATTCTGCTAACT *CP4EPSPS*: AAACATGAAGGACCTGTGGGAGATAGACTTGTCACCTGGAATACGGACGGTTCCAGAAAGACCAGAGGACTTACGAGCAGT
L_oaxwhite	*Cry2Ab*: CTCCCTTATCGGTTTCTCTGAAGACGCAGCAGCATCCACGAGCTTATCCGATTTCGTCGTCGTGGGGATTGAAGAAGAGTGGGATGACGTTAATTGGCTCTGAGCTTCGTCCTCTTAAGGTCATGTCTTCTGTTTC *CP4EPSPS*: ACTGCTCGTAAGTCCTCTGGTCTTTCTGGAACCGTCCGTATTCCAGGTGACAAGTCTATCTCCCACAGGTCCTTCATGTTT

**FIGURE 2:**
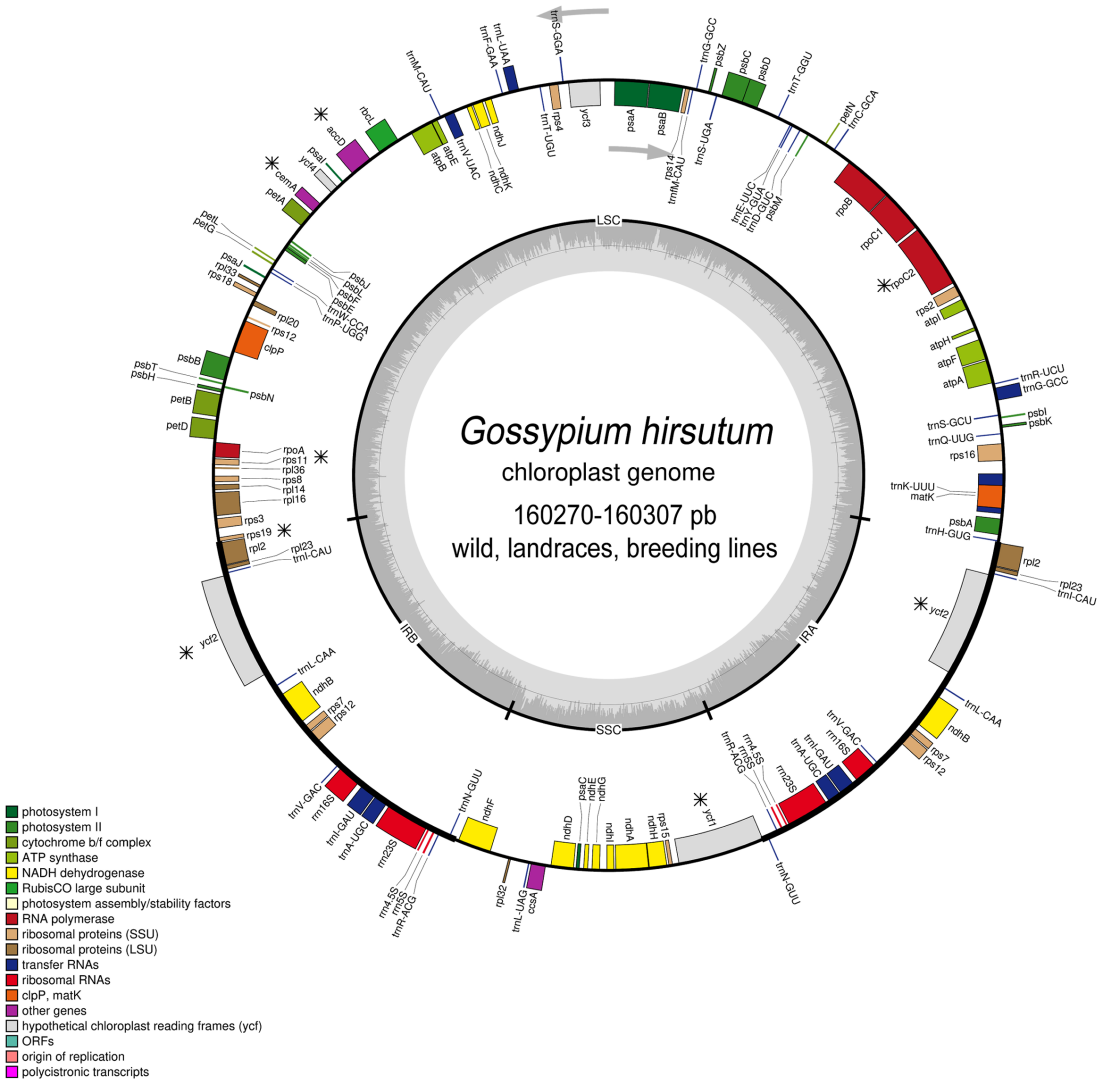
Circular map of the chloroplast genome of *Gossypium hirsutum* with annotated genes. The genes transcribed clockwise are shown inside the circle, and the genes transcribed counterclockwise are shown outside the circle. The dashed gray color of the inner circle shows the GC content. The color of genes is related to the functional categories in the legend. Genes with selection signals are marked with an asterisk.

**FIGURE 3:**
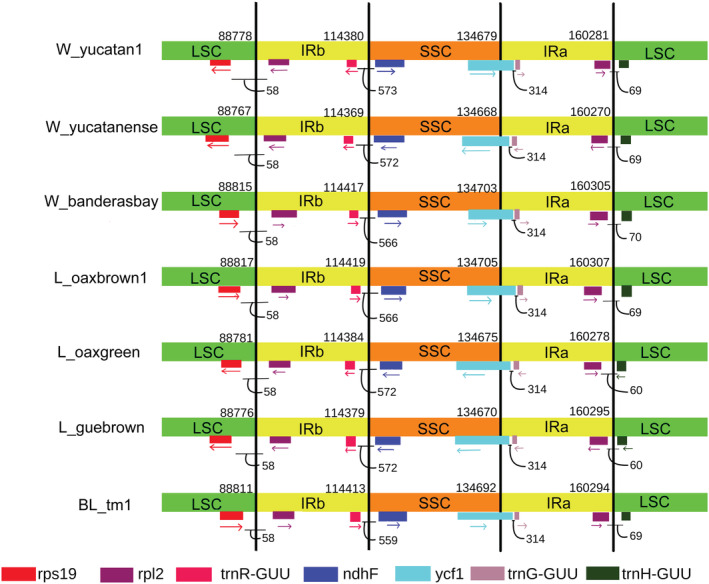
Comparison of the LSC, SSC, and IRs borders among wild and domesticated genomes with more than 2 bp in the length of contraction/expansion. The variability in contraction/expansion cannot be associated with domestication processes; however, the BL genomes are homogeneous. The arrows indicate the movement of the position of genes compared between them; numbers under genes are distances (bp) between genes from the borders. Conjunctions IRb/SSC and IRa/LSC exhibit more variability and *ycf1* is the only gene that crosses the border.

We assembled and annotated 23 new chloroplast genomes of wild, landrace, and BL *G. hirsutum* samples. All samples shared the four typical structures of the organelles: inverted repeats (IR; 25,601–25,602 bp long), large single copy (LSC; 88,767–88,817 bp long), and small single copy (SSC; 20,278–20,298 bp long), with an identical arrangement and 37% of CG content. The same gene composition of 131 genes was preserved, just as in the available *G. hirsutum* plastomes, although the assemblies featured an inversion in the SSC region with respect to reference genome Coker 310 in all samples (Figure 2). The chloroplast length ranged between 160,270 and 160,307 bp, yet wild genomes were slightly shorter than domesticated cotton (Table 1). There was a slight variation in the border junction of the LSC‐IRb‐SSC‐IRa: between 2 and 50 bp (Figure 3). The border's expansion can be explained by the modifications in intergenic regions, mainly in the IRb/SSC and IRa/LSC borders, and there was no partial loss in any of the genes.

The Mauve alignment algorithm corroborated the absence of structural variants and showed the homologous regions shared in each component of the wild‐to‐domesticated complex (Figure S1a). The lime block between 148,100–148,498 positions turned out to be unique to wild genomes and was associated with the *ndhB* gene. In domesticated plastomes, the region between 142,091–143,785 (blue and green blocks) was homologous in the *rrn16S* gene. Within the domesticated genomes, we found particular regions, such as the mint green block (9661–13,555 bp), shared by the landraces of Guerrero and Oaxaca (green variety cotton), which correspond to the *trnG‐GCC* gene (Figure S1).

The selection effects in wild, landrace, and BL genomes were different according to the Tajima's D values: −1.74084 (*p*‐value < .05), −0.24844 (*p*‐value > .10), and 1.22474 (*p*‐value > .10), respectively (Figure S2). Particularly in the borders junction of the LSC‐IRb‐SSC‐IRa where selection signals are frequently noted, we identified a potential selection signal in the *ycf1* gene (*D* = −1.05 *p*‐value > .10; SSC‐IRa border). In addition to the *ycf1* gene, we identified *rpoC2*, *ycf2*, *clpP*, *cemA*, *rps11*, and *accD* genes under selection through the Tajima’ D test, and they were therefore candidates to test the ratio of nonsynonymous to synonymous substitutions (dN/dS) with PAML. The complete results obtained in each comparison are shown in Table 3. In general, the genes were undergoing a purifying selection (dN/dS < 1), except in *ycf1*, which was under positive selection in genome landraces (dN/dS > 1).

**TABLE 3:** Test of selection (neutral, purifying, positive) in sequence pairs of wild, landraces, and breeding line genes.

Gene	Comparison among wild dN/dS	Comparison among landraces dN/dS	Comparison among BL dN/dS	Comparison wild‐landraces dN/dS	Comparison wild‐BL dN/dS	Comparison landraces‐BL dN/dS
*rpoC2*	–	–	–	0.3998	0.3998	0.3998
*ycf2*	–	0.4939	–	0.4946	0.4350	0.1473
*ycf1*	–	4.3220	–	0.2081	0.2079	0.2070
*clpP*	–	0.2481	0.2231	–	–	0.3089
*accD*	–	–	0.4623	0.3851	0.4509	–
*cemA*	–	–	0.2829	0.1126	–	0.4215
*rps11*	0.2630	–	–	–	–	–

*Note*: The middle dash indicates when dN/dS is indeterminate. The ratio is often used as a measure of selective pressure indicating neutral substitution rates (dN/dS = 1), positive (dN/dS > 1), and purifying selection (dN/dS < 1), respectively.

Using the chloroplast reference genome of *G. hirsutum* Coker 310 FR we identified 1407 SNVs, 304 Indels, and 197 STRs. The sums of the genomic variants found (SNV, STR, and Indels) represent 44.39% in wild genomes, 34.27% in local varieties, and 21.33% in BL genomes, of the total genomic diversity of the wild‐to‐domesticated complex of cotton. It is worth mentioning that genomes of transgene‐introgressed samples showed less diversity than the genomes from samples of the same variety or the samples obtained from the same wild population without transgenes. Particularly, brown landraces genomes from Oaxaca with transgenes showed less variations than Yucatán's genomes with transgenes: 21.85%–51.27% of variation reduction in wild genomes of TI samples regarding chloroplast genome without transgene sample; and 85.16%–89.85% reduction in landraces genomes of TI samples regarding chloroplast genome without transgene sample (Table 4). After removing variants with <20% missing calls and minor allele frequency (MAF) > 0.05, the number of SNPs in each subgroup was 131 for wild, 57 for landraces, and 76 for BL. The number of Indels and STRs were: 17, 40 in wild; 14, 24 in landraces; and 27, 4 in BL after filtering. Mostly, genomic variants were concentrated in the *ycf1*, *rpl32*, *psbJ*, *rps16*, *rps4*, *atpI*, and *atpH* genes. However, between the wild‐to‐domesticated complex, the genes with more genomic variants (SNPs, Indels, and STRS) were *ndhD*, *matk*, *petN*, *psbZ*, and *rps14* in wild; *atpA*, *atpF*, *psbC*, and *psbL* in landraces; and *rpl16* and *ndhG* in BL (Table S2).

**TABLE 4:** Summary of genomic variants in plastomes of wild, landraces, and breeding lines cotton (*Gossypium hirsutum*).

Sample	Domestication status	Indels	SNVs	STRs	Total
W_yucatan1	Wild	15	93	11	119
**W_yucatan2**	**Wild**	**13**	**64**	**14**	**91**
**W_yucatan3**	**Wild**	**11**	**47**	**13**	**71**
**W_yucatan4**	**Wild**	**11**	**34**	**13**	**58**
**W_yucatan5**	**Wild**	**12**	**68**	**13**	**93**
W_yucatanense	Wild	33	175	17	225
W_banderasbay	Wild	8	63	10	81
W_guatemala	Wild	23	73	13	109
L_oaxbrown1	Landraces	11	109	8	128
**L_oaxbrown2**	**Landraces**	**5**	**10**	**4**	**19**
**L_oaxbrown3**	**Landraces**	**4**	**5**	**4**	**13**
**L_oaxbrown4**	**Landraces**	**6**	**6**	**3**	**15**
**L_oaxbrown5**	**Landraces**	**10**	**3**	**3**	**16**
L_oaxgreen	Landraces	14	100	13	127
**L_oaxwhite**	**Landraces**	**2**	**14**	**5**	**21**
L_guebrown	Landraces	13	109	12	134
L_guegreen	Landraces	13	54	11	78
L_guewhite	Landraces	15	77	11	103
BL_tm1	Breeding lines	22	125	5	152
BL_stoneville474	Breeding lines	14	51	4	69
BL_Coker312	Breeding lines	18	27	4	49
BL_fibermax832	Breeding lines	21	63	4	88
BL_acalamaxxa	Breeding lines	10	37	2	49

*Note*: Samples in bold indicate individuals with the presence of transgenes.

**FIGURE 4:**
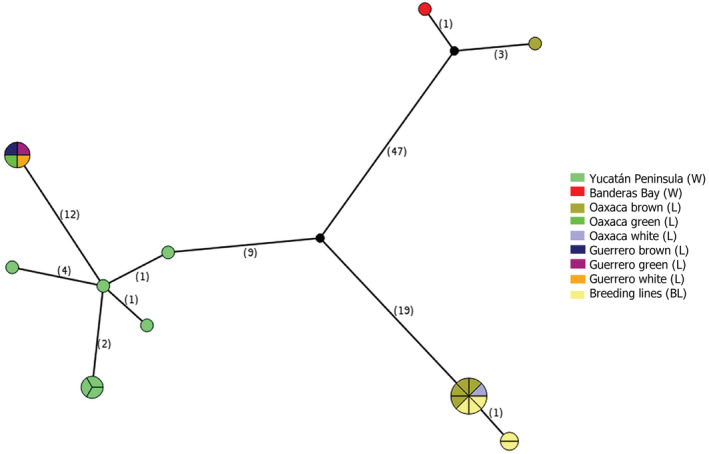
Haplotype network analysis. Median‐joining network based on CDS regions. Each ball represents a haplotype, the size of which is proportional to its overall frequency in the wild‐to‐domesticated cotton complex. The number in parentheses indicates the number of mutational changes between two different haplotypes, and black dots correspond to missing haplotypes. The Yucatán Peninsula contains the highest diversity of haplotypes within the analyzed dataset. Wild individuals analyzed present exclusive haplotypes, compared with samples from domesticated.

The nucleotide diversity of all genomes was low (π = 0.00016), but landraces exhibited the highest diversity (π = 0.00018), followed by wild populations (π = 0.00011), while BL showed the lowest Pi value (π = 0). When the chloroplast from TI individuals was separated, the values of nucleotide diversity were 0.00020, 0.00001, 0.00016, 0, and 0, for wild, TI‐wild, landraces, TI‐landraces, and BL, respectively. The BL haplotype diversity was equal to 0.6 ± 0.175 with 2 haplotypes; the landraces´ was *H*_d_ = 0.644 ± 0.101 and 3 haplotypes; and the wild's haplotype diversity reached *H*_d_ = 0.893 ± 0.111 and 6 haplotypes. When we separated the TI samples, we obtained the following results: *H*_d_ = 1.00000 (4), 0.50000 (2), 0.40000 (2), 0.00000 (1), 0.60000 (2) of wild, TI‐wild, landraces, TI‐landraces, and breeding lines, respectively; the number of haplotypes is indicated in brackets. In the haplotype network, it is possible to see which haplotypes are shared between the groups with different degrees of management (Figure 4). The population structure analysis showed that the optimal number of clustered groups was four: Yucatán Peninsula; Banderas Bay together with L_oaxbrown1, L_oaxbrown2, and L_oaxbrown3; the rest of the samples of landraces from Oaxaca and breeding lines; and landraces from Guerrero. The result of the Bayesian analysis was congruent with the analysis of the relation of 10 haplotypes that were found in all samples.

Using the N_ST_ approach, the higher differentiation was found between wild populations and BL (*N*_ST_ = 0.75070), while the pairwise values for landraces generally showed low differentiation compared to the differences between other groups: (*N*_ST_ value 0.29755 with BL, and 0.26917 with wild populations). The calculation of the *N*_ST_ value distinguishing the TI samples shown in Table S3 determined that the genetic difference between wild and TI‐wild was higher than that between wild populations and landraces.

### 3.2 Evolutionary relationships of the wild‐to‐domesticated complex of cotton and among the *Gossypium* species

**FIGURE 5:**
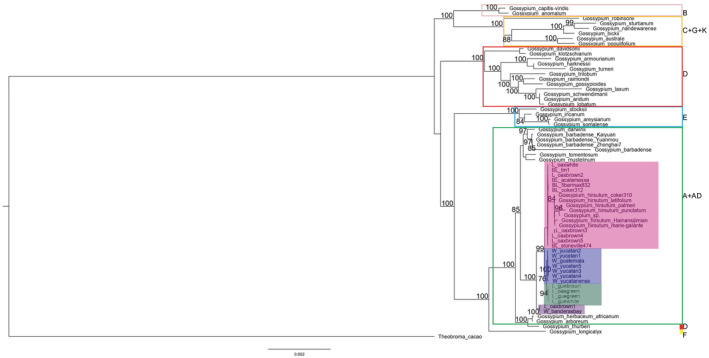
Phylogenetic tree of Gossypium inferred using RAxML from SSC dataset. Colored rectangles frame the genomic clades and shading color block marked the wild‐to‐domesticated cotton complex, pink shows the BL, Hutchinson's landraces and TI‐landraces clade, wild clade in blue, landraces clade in green, and wild+landraces in purple.

In order to develop the tree topologies, we used SSC, LCS + IR, and the whole chloroplast dataset. In the LCS + IR tree, the lower cluster showed the well‐known phylogenetic topology of *Gossypium* genus, clustered by genome type: A + AD, F, D, B, and C + G + K. Our samples were grouped within the A + AD genome node (85 bootstraps value) (Figure 5). The closest samples to the AD cluster were *G. arboreum* (genome type A), *G. herbaceum* subsp. *Africanum* (A), and *G. thurberi* (D), which have been considered as the possible chloroplast donor species. Our samples were grouped into four different phylogenetic clusters with high support values: (1) BL + TI‐brown and TI‐white landraces from Oaxaca + Hutchinson's varieties; (2) wild samples; (3) landraces; (4) wild + landraces (84, 99, 100, 94 and 100 bootstraps values). Both the SSC and the whole chloroplast tree showed the same topology: two main polyphyletic clusters with high support formed by different genomic types, caused by the high diversity in the SSC region (Figure S3). Unlike the LCS + IR tree, the *G. thurberi* was closer to the AD cluster while *G. arboreum* and *G. herbaceum* subsp. *africanum* was closer to the A + AD cluster.

**FIGURE 6:**
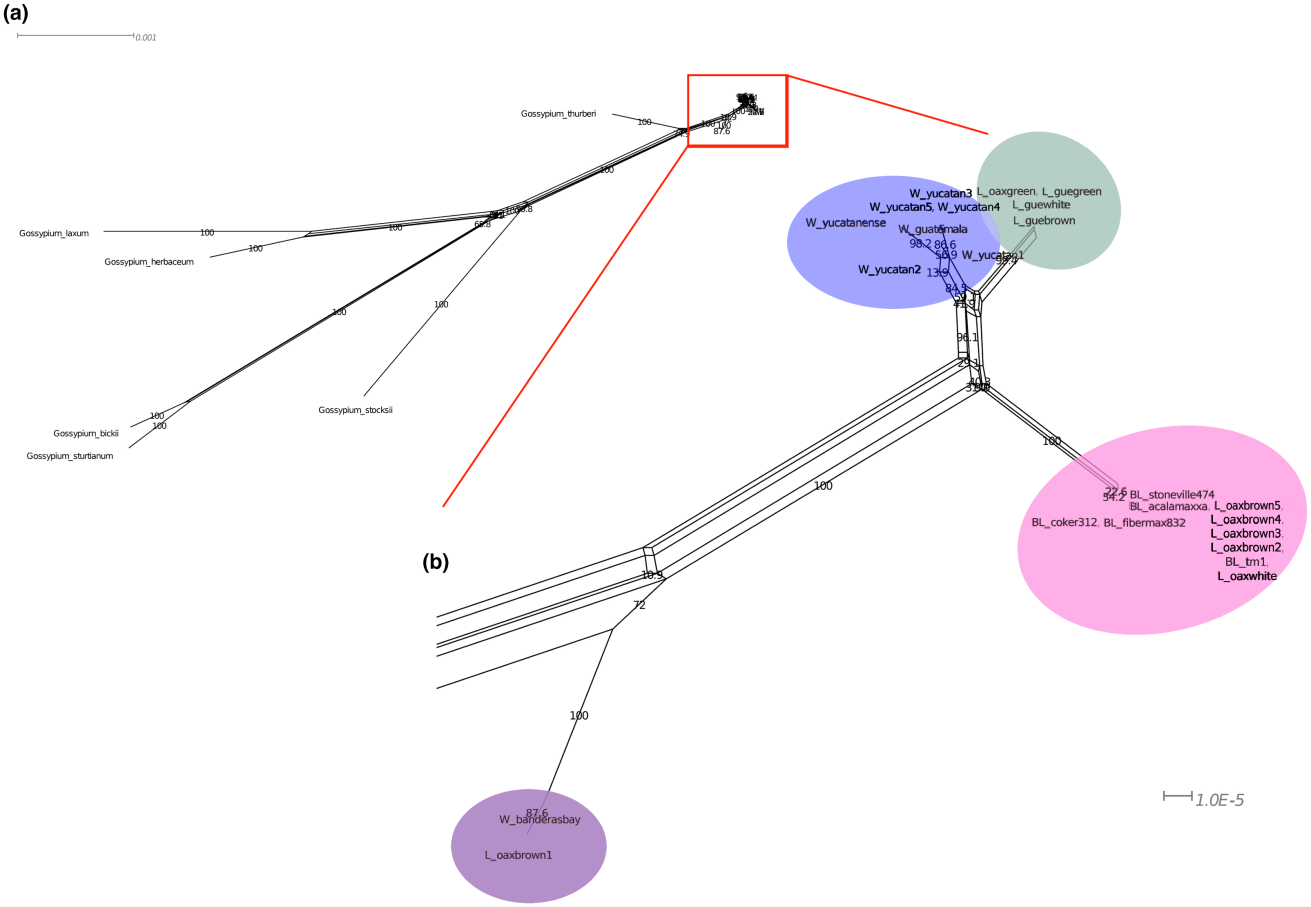
(a) Phylogenetic network constructed with the LSC + IR dataset, the outgroup species was formed with a representation of each genome type of Gossypium. (b) Relationships among our samples in four main groups. The length of the branches showed the divergence between wild and domesticated G. hirsutum. Scale marks the substitutions per site and the number of branches is bootstrap support. Shading color ovals marked the wild‐to‐domesticated cotton complex, pink shows the BL and TI‐landraces clade, wild clade in blue, landraces clade in green, and wild+landraces in purple.

All chloroplasts were grouped within the rightmost of the phylogenetic network (Figure 6a). Just as in the phylogenetic tree, four different clusters were formed: W_banderasbay and L_oaxbrown1 grouped far away from the rest of the groups; landraces (green from Oaxaca and Guerrero; white and brown from Guerrero) were close to the wild samples (Yucatán and Guatemala); and the remaining landraces from TI samples (brown and white from Oaxaca) were clustered with the BL samples. Banderas Bay and brown from Oaxaca clustered together near the wild samples but at different borders. Again, *G. thurberi* was closer than *G. herbaceum* to the samples of the cotton's wild‐to‐domesticated complex.

**FIGURE 7:**
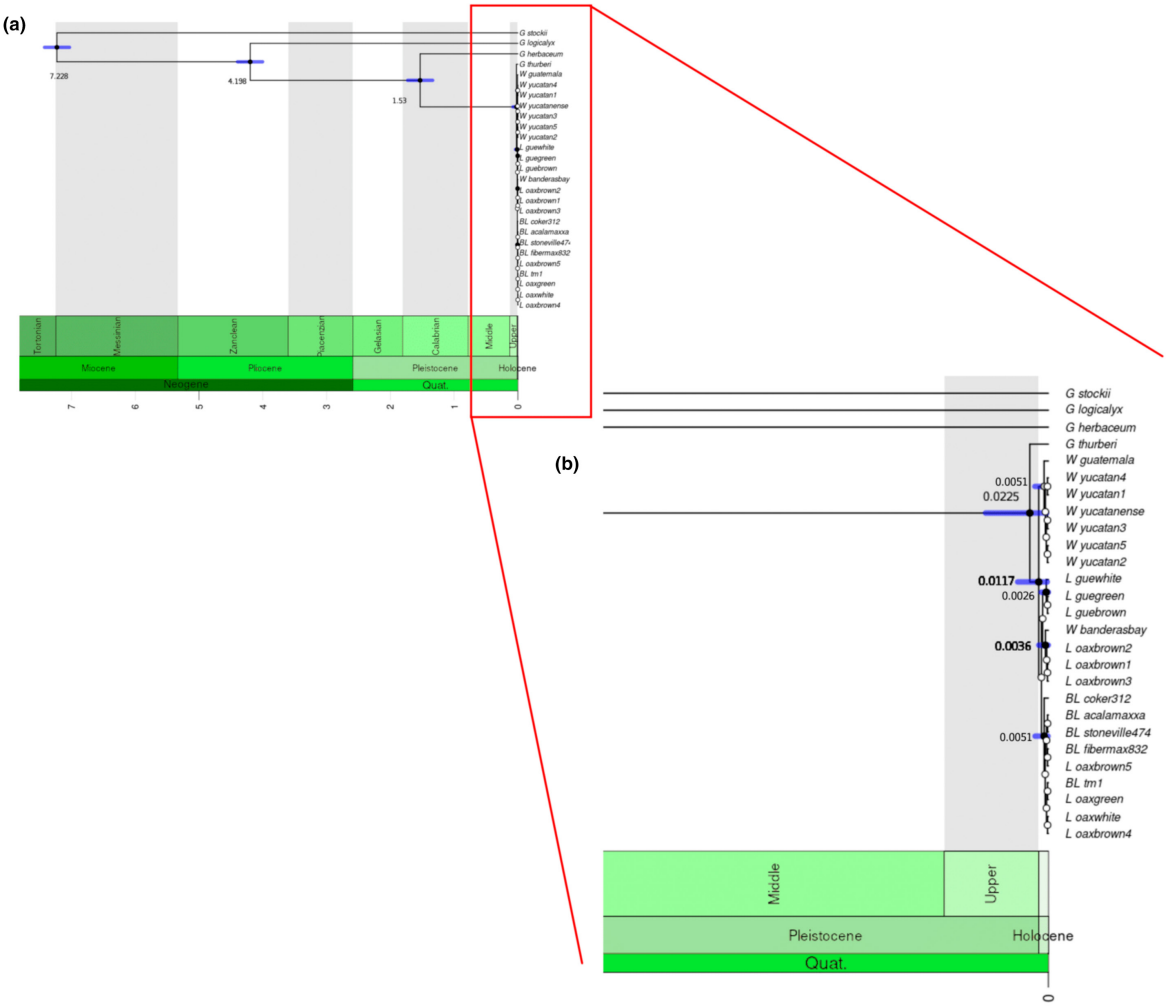
Phylogenetic tree inferred from CDS regions showing the divergence times A: between Gossypium species and the samples of wild to domesticated complex of cotton; B: zooming up the Gossypium hirsutum clade, bold numbers are the divergence times between wild and domesticated. The posterior probability is represented with the point color, blank is ≥ 0.95 and white is < 0.95 posterior probability. Numbers close to the nodes are the divergence time in millions of years before the present (time scale is mya); blue bars correspond to 95% of confidentiality interval of the mean time to the common ancestor.

Figure 7a displays the divergence times between wild populations and crops using the coding sequences and the calibration developed based on the divergence times estimated among *Gossypium* genomic groups. The divergence time estimated between the wild variety from Yucatán‐Guatemala and the rest of the samples was 0.0117 mya (11,700 years BP), while the time between W_banderasbay and coyuchi from Oaxaca was less ample: 0.0036 mya (3600 years BP; Figure 7b).

## 4 DISCUSSION

### 4.1 Structure and homologous regions that characterize wild and domesticated plastomes

Domestication has not determined the structural modifications of the chloroplast. However, in our assemblies, the inversion of the SSC region could be associated with a double structural haplotype present in the species, as recorded in the *G. barbadense* and the *G. thurberi* (Ibrahim et al., 2006; Talat & Wang, 2015; Wang & Lanfear, 2019).

We also observed an increase in the chloroplast length of domesticated cotton in intergenic regions, mainly in the LSC and SSC regions related to the variability at the borders. However, the variation in the LCS‐IR‐SSC‐IR borders cannot be associated with selection by domestication because it occurs in all plastomes, although the BL genomes are less variable (Figure 3). The contraction/expansion in the size and changes on borders of chloroplast was frequent in all plant species due to selection pressures and phylogenetic signals (He et al., 2020; Xiao‐Ming et al., 2017). Domestication involves a selection pressure that can accelerate the contraction/expansion and modify the genome size. Just as in cotton, Muraguri et al. (2020) observed an increase in the length of chloroplast in *Ricinus communis* domesticated. Contrarily, a contraction of genomes was identified in domesticated plants in *Solanum lycopersicum* (Tamburino et al., 2020).

When comparing the homologous regions between genomes, we identified patterns that distinguish the wild ones from the domesticated ones, exhibiting the differences in their evolutionary pathways. Often, information from morphological traits and distribution is confusing and makes it hard to distinguish between wild and domesticated plants, which is why we sought for both particular and variable markers (Nock et al., 2019). The *ndhB*, *rrn16S*, and *trn*G‐GCC genes could work as particular markers to distinguish domesticated from wild cotton. *dhB* is a transmembrane gene with translocase function involved in photosynthesis under humidity stress conditions (Horváth et al., 2000); *rrn16S* is a cellular component of the ribosomal subunit with high diversity, e.g., in *Bulbophyllum* (Tang et al., 2021); and *trn*G‐GCC is a transfer RNA mentioned as a variable and useful gene for taxonomic identification in Acanthaceae and Balsaminaceae, it is also used to analyze the effects of domestication in *Chimonanthus praecox* (Huang et al., 2020; Lu et al., 2015; Luo et al., 2021).

### 4.2 Genes under selection through domestication

It is possible to identify different selection signals among crops and wild relatives associated with variable environments and domestication, since (1) high productivity may be related to an increase in energy demand, as observed in wheat crops; (2) wild and domesticated plants are commonly under different management; (3) the chloroplast of wild relatives must meet the energy demand to survive various environmental conditions (Aliyev, 2012; Dusenge et al., 2019). The negative Tajima's *D* values found here suggest that wild and landraces were under a recent selection process with a tendency to population expansion. However, the selection was unequal for both wild and landraces (wild *D* = −1.74084 *p*‐value < .05; landraces *D* = −0.24844 *p*‐value > .10). On the contrary, BL showed signs of a balancing selection where the most common phenotype tends to be preserved (*D* = 1.22474 *p*‐value > .10).

The genes that showed selection signals had most of the genomic variants. Thus, they could be potential candidates for molecular markers in future research. They are involved in diverse cellular functions: *rpoC2* partakes in the transcription process (Tadini et al., 2020); *clpP* cleaves peptides into proteins in a process that requires ATP hydrolysis (Majeran et al., 2019); *accD* presumably takes place in fatty acid biosynthesis (Bryant et al., 2011); *cemA* is involved in transmembrane proton transporter activity (Park et al., 2019); *rps11* is implicated in the translation process and the viral interactions (Wang et al., 2017); *ycf1* and *ycf2* (possible ATPasa) are genes with unknown functions in the chloroplast genome, but they are essential to protein import into chloroplast stroma (Drescher et al., 2000; Kikuchi et al., 2013). Most of these genes were under a purifying selection due to nonsynonymous mutations that could be harmful to the production of energy processes (Du et al., 2020; Wang, Zhou, et al., 2018; Xu et al., 2015). Only the *ycf1* gene showed positive selection among landraces, as it has been observed in species like *Citrus* genus (Carbonell‐Caballero et al., 2015). This could be an indicator that native varieties are preserving new alleles that allow them to survive in different environments.

### 4.3 Domestication and introgression modify the genetic diversity into wild‐to‐domesticated cotton complex

Human selection operating through plant domestication commonly leads to a reduction in genetic diversity (Smýkal et al., 2018). Here, wild populations contained more diversity than domesticated stands, mainly in the number of STRs (Table 4). This is consistent with the reports provided by Wegier et al. (2011). Furthermore, genetic diversity in landraces did not decrease drastically as in the BL. It is worth considering that the landrace samples included white, brown, and green varieties from two Mexican regions. The domestication processes that helped create these varieties might increase the genetic diversity, as reported in diversity comparisons in chili peppers, pumpkins, and bean landraces (Andueza‐Noh et al., 2013; Castellanos‐Morales et al., 2019; Castilla et al., 2019; Félix et al., 2014). Expression of management, together with ecological processes and specific phenotype selection played an important role in the preservation of diversity. For instance, women from indigenous communities who produce textiles, spin and weave cotton by hand so that they are able to choose the seeds with better quality for the next agricultural cycle. Parallelly, cottonseed exchange is a recurrent practice, and the pollination of cotton occurs uncontrolled. In accordance, the phylogenetic network and the *N*_ST_ values reflected the continuous gene flow between wild and landraces populations, and between landraces and BL. In addition, the presence of transgenes (*Cry1Ab/Ac*, *Cry2Ab*, and *CP4EPSPS*) in wild and landraces can be considered as markers that exhibit the recent and continuous gene flow and introgression.

The introgression of domesticated alleles and transgenes is considered a factor that modifies the genetic diversity of the nuclear genome (Ellstrand et al., 2002; Rojas‐Barrera et al., 2019; Wang et al., 2019). Interestingly, plastomes of transgenic samples showed less diversity and according to RhierBAPS, haplotype network, and phylogenetic analysis (Figures 4 and 5) landraces plastomes shared ancestor with the BL, which is not the case in wild plastomes with transgenes. The introgression of transgenes in the wild‐to‐domesticated cotton complex could succeed via pollen and through the transgenic seeds' establishment and subsequent pollination. But the possible effect on diversity will depend on whether the donor of the chloroplast genome to the next generation is transgenic or nontransgenic as described in Figure S4.

The study of the diversity of plastomes through the introgression of domesticated alleles or transgenes is scarce because it is a nonrecombinant genome and the *Agrobacterium*‐mediated transformation through which transgenes are inserted is directed to the nuclear genome (Tzfira & Citovsky, 2006). However, nuclear and cytoplasmic genomes are highly dynamic, and the transcriptional activity of chloroplast requires coordination with the nuclear gene expression, for instance, the interplay of plastid‐encoded polymerases and nuclear‐encoded polymerases is necessary for the correct development of functional chloroplast (Tadini et al., 2020). Therefore, nuclear or cytoplasmic gene changes could have indirect repercussions among them. Furthermore, diversity or gene expression modifications by transgenes could have phenotypic alterations such as propagation, leaf production, and height increase rate; microbial growth; and root development in cotton culture in vitro, which can translate into the distinction of metabolic costs (Hernández‐Terán et al., 2019). On the other hand, it is possible that the changes that we observed are related to the introgression of domesticated alleles and the diversity reduction related to domestication processes. Inevitably, the genetic diversity in transgenic crops is lower than in conventional crops, since the transgenic varieties are developed through pure line cells (Kumria et al., 2003).

### 4.4 Multiple events of domestication generated cotton landraces in Mexico

Various molecular markers have been used to elucidate *Gossypium*'s evolutionary history and chloroplast has proven to be the most reliable for resolving its uncertainties, due to its maternal inheritance (Chen et al., 2016, 2017; Wu et al., 2018). However, the presence of two structural haplotypes with respect to the SSC region, added to include high variability regions, can generate discrepancies in the topologies (Figure 7 and Figure S3). Based on the less variable regions, we reconstructed a phylogeny similar to the previously reported ones, in which *G. herbaceum*, *G. arboreum*, and *G. thurberi* are the closest taxa to the AD clade (Figure 7; Wu et al., 2018; Xu et al., 2012). In addition, the phylogenetic analysis supported the identity and occurrence of the wild populations described by Wegier et al. (2011) and Alavez et al. (2021).

The domestication of *G. hirsutum* originated in Mesoamerica. More exactly, the hypothesis proposed by Brubaker and Wendel (1994) suggested that domestication began through the selection of wild populations from the Yucatán peninsula. In order to do this, they took into account Hutchinson (1951), Fryxell (1979), and Stephens (1958) classifications, in which *yucatanese* corresponds to a wild race of cotton considered *truly wild*. It was from this taxon, according to these authors, that the *punctatum*, *palmeri*, and *latifolium* varieties emerged, which are recognized as wild or agronomically primitive forms, from which BL was subsequently obtained (Figure 1h). The phylogenetic tree in this study revealed that wild and landraces clades were sister nodes of BL + TI‐landraces + Hutchinson's varieties. This result was concordant with Zhang et al. (2019) who proposed that the closest relationship of *G. hirsutum* BL was the *latifolium*, *richmondi*, and *marie‐galante* varieties whereas its most distant relationship was the *yucatanense* variety, which clearly corresponds to a wild individual from the Yucatán Peninsula metapopulation.

Additionally, the results provided evidence that genetic resources from the Yucatán Peninsula metapopulation (including the Guatemalan distribution area) were used in the development of BL, and that landraces played an important role in the domestication process. Unlike trees, the phylogenetic network suggested more than one evolutionary history. Although the divergence between wild and BL was lower than BL and landraces, alternate branches did not reject any alternative topology. The position of W_banderasbay and L_oaxbrown as the basal node in the tree shown in Figure 7 and the divergence observed in the network indicated multiple domestication events. It has been observed that the Banderas Bay holds higher haplotype and morphological diversity (e.g., form of leaf, fiber characteristics, and foliar epidermal characters) than the rest of metapopulations (Uscanga, 2013; Vega, 2015; Wegier et al., 2011). This pattern suggested the need of expanding the analysis to the Center and North Pacific regions and increase the number of samples of all metapopulations, since these could provide more information regarding cotton's domestication events.

The genetic divergence and the evidence of introgression observed match with the archeological, historical, and ethnobotanical records. Regarding the multiple origins of landraces, the women weavers from Guerrero corroborated our results; they consider that the fiber obtained from cotton from Guerrero, Oaxaca, and Veracruz have different characteristics among them, which is why they need to undergo different procedures before spinning them. Another evidence of multiple domestication events that helps calculate the time in which the cotton domestication began in Mexico is the finding of cotton archeological remains. These have been found in Tehuacán, Puebla and dated between 5500–4300 years BP; in the Valley of Oaxaca in a context dated 1300 years BP (Smith & Stephens, 1971); and in the Balsas region (dated in 1200 years BP). The divergence time estimated from coding regions of chloroplast suggested that the domestication could have started 11,700 years ago (Bjerregaard & Peters, 2017). However, there is a variation range in the node calibration shown with the blue bar (Figure 6).

### 4.5 Implication to conservation of wild population and landraces in Mexico

The Convention on Biological Diversity supported by the Nagoya protocol and the Mexican law: *Ley de bioseguridad de organismos genéticamente modificados*, promoted the application of strategies for the preservation of biological diversity. Also, the sustainable use of its components and the fair and equitable sharing of the benefits arising from the utilization of genetic resources, prioritizing the centers of domestication, origin, and diversity where the crop wild relatives are distributed (Diario Oficial de la Federación, 2005; Secretariat of the Convention on Biological Diversity, 2011). Under such premise, and considering cotton's risk category (vulnerable), the results shown in this research should be integrated into the public policies about cotton genetic resources in Mexico and the benefits shared with indigenous and local communities.

The low diversity observed in the chloroplast, either by the introgression of domesticated alleles or transgenes, is concerning because it limits its capability to respond to environmental changes. Considering the development of transgenics directed to the chloroplast (in cotton and other crops; Daniell et al., 2005; Kumar et al., 2004), from biosafety, we suggest the expansion of the analysis about the interaction between nuclear and cytoplasmic genomes, together with the understanding of the frequency and accumulation of introgression, as well as the consequences according to the introgressed transgene (Vázquez‐Barrios et al., 2021). At this point, it is necessary to clarify that the inclusion of TI samples from Oaxaca (white and brown) and Yucatán in this study does not mean that the rest of the varieties and populations are out of gene flow and introgression processes. Gene flow and introgression occur at different frequencies, so integral work must be done with the communities to monitor and mitigate environmental and cultural consequences, as well as to plan adequate policies for the conservation of agrobiodiversity.

Using only chloroplast limits the differentiation between introgression events that occurred in the past from recent events, therefore we consider that further research should include the chloroplast of the rest of metapopulation, more samples of landraces; and should incorporate nuclear genome markers. The aforementioned actions will allow detailing evolutionary history; recognizing the origin of the genetic material used in crops and research throughout the world; and providing information to describe the transgenes dispersion routes (Alavez et al., 2021). This information is key for sharing the benefits and avoiding harm to the local and indigenous communities, which are fundamental to the domestication processes and to the protection of genetic resources.

## AUTHOR CONTRIBUTIONS

**Melania Vega:** Conceptualization (lead); data curation (lead); formal analysis (lead); investigation (lead); methodology (lead); project administration (lead); software (lead); supervision (lead); validation (equal); visualization (equal); writing – original draft (lead); writing – review and editing (lead). **Christian Quintero‐Corrales:** Data curation (supporting); formal analysis (supporting); methodology (supporting); software (lead); validation (supporting); visualization (supporting); writing – original draft (equal); writing – review and editing (equal). **Alicia Mastretta‐Yanes:** Conceptualization (supporting); investigation (supporting); supervision (equal); writing – original draft (supporting); writing – review and editing (equal). **Alejandro Casas:** Conceptualization (supporting); investigation (supporting); supervision (equal); writing – original draft (supporting); writing – review and editing (equal). **Victorina López‐Hilario:** Investigation (supporting); supervision (equal); writing – review and editing (equal). **Ana Wegier:** Conceptualization (lead); funding acquisition (lead); investigation (equal); methodology (equal); project administration (lead); resources (lead); supervision (lead); validation (equal); visualization (equal); writing – original draft (equal); writing – review and editing (equal).

## BENEFIT SHARING

The benefits of this research include the generation of information on local varieties requested by local communities. The results were communicated to collaborators in the local communities, and their comments have been included in the discussion. In addition, they are recognized in the document as authors or in the acknowledgments according to their personal decision.

## Supporting information


Data S1

## Data Availability

Assemblies sequences were deposited to NCBI. Vcf files associated are available in: https://www.researchgate.net/publication/358977040_chloroplast‐vcftar All code used to perform our analyses and figures are available: https://github.com/conservationgenetics/Multiple_domestication_events_explain_the_origin_of_landraces_of_Gossypium_hirsutum_in_Mexico.git
