# Supplementary material for: Multiple domestication events explain the origin of Gossypium hirsutum landraces in Mexico
Source: Ecol Evol. 2023 Mar 8;13(3):e9838. doi: 10.1002/ece3.9838 (PMC9994486; doi:10.1002/ece3.9838)
Supplement: Supplementary file 1 — Data S1 [file ECE3-13-e9838-s001.pdf]

Supplemental Information for:

Multiple domestication events explain the origin of *Gossypium hirsutum* landraces in Mexico

Melania Vega, Christian Quintero-Corrales, Alicia Mastretta-Yanes, Alejandro Casas, Victorina López-Hilario, Ana Wegier

Table S1. Characteristics of the available genomes that were used to analyze phylogenetic relationships.

Species	Genome group	Total	LSC	SSC	IR	Accession ID
G. herbaceum africanum	A	160315	88790	20285	25620	NC_016692
G. arboreum		160230	88722	20274	25617	NC_016712
G. anomalum	B	159507	88102	20199	25602	NC_023213
G. capitis-viridis		159467	88065	20198	25602	NC_018111
G. sturtianum	C	159627	88251	20216	25580	NC_023218
G. robinsonii		159726	88359	20203	25582	NC_018113
G. nandewarense		159677	88284	20241	25576	MG779276
G. thurberi	D	160264	88737	20271	25628	NC_015204
G. harknessii		160129	88710	20221	25599	NC_033333
G. davidsonii		160027	88628	20240	25602	NC_033395
G. klotzschianum		160097	88654	20235	25604	NC_033394
G. aridum		160257	88720	20243	25647	NC_033396
G. raimondii		160161	88654	20205	25650	NC_016668
G. gossypioides		159959	88803	20004	25576	NC_017894
G. armourianum		160080	88657	20241	25591	MG891801
G. lobatum		160205	88811	20294	25550	MG891802
G. trilobum		160142	88735	20233	25587	MG800783
G. schwendimanii		160199	88779	20318	25551	MG891803
G. laxum		159947	88582	20240	25683	KF806549
G. turneri		159927	88412	20215	25650	NC_026835
G. stocksii	E	159039	87886	20179	25487	NC_023217
G. somalense		159539	88150	20251	25569	NC_018110
G. areysianum		159572	88182	20252	25569	NC_018112
G. incanum		159205	87879	20196	25565	NC_018109
G. longicalyx	F	160241	88667	20278	25648	NC_023216
G. bickii	G	159422	88073	20183	25583	NC_023214

G. australe		159578	88223	20221	25567	NC_033401
G. populifolium	K	159444	88197	20093	25577	NC_033398
G. hirsutum latifolium	AD	160347	88848	20287	25606	MG800784
G. hirsutum Hainansijimian		160265	88782	20279	25602	HQ901197
G. hirsutum		160301	88816	20269	25608	NC_007944
G. barbadense		160317	88841	20294	25591	NC_008641
G. barbadense Zhonghai		160302	88849	20267	25593	HQ901199
G. barbadense Yuanmou		160291	88838	20267	25593	HQ901198
G. barbadense Kaiyuan		160291	88836	20267	25594	HQ901200
G. tomentosum		160433	88932	20271	25615	NC_016690
G. mustelinum		160313	88826	20269	25609	NC_016711
G. darwinii		160378	88906	20266	25603	NC_016670

Table S2. Genes modified by genomic variants in the wild-to-domesticated cotton complex. The highlighted words correspond to genes that have genomic variants only in that part of the wild-to-domesticated complex.

	SNPs	Indels	STRs
Wild	*psbA*, ***matk***, *rps16,atpI*, *rpoC2*, *rpoB*, ***petN***, ***psbZ***, *ycf3*, *psaI*, *petA*, *psbJ*, *rpl33*, *rps18*, *petD*, *psaA*, *rpoA*, ***rps11***, ***rps14***, ***rps3***, *rps19*, ***ndhE***, *ccsA*, *rpl32*, *ndhF*, *ycf1*	*psbA*, *rps16*, *atpH atpI*, *rpoC2*, *rpoB*, *ycf3*, *rps4*, *ndhK*, *atpB*, *rbcL*, *psaI*, *cemA*, *psbJ*, *psaJ*, *rpl33*, *rps18*, *rpl36*, *rps19*, *ycf1*, ***ndhD***, *ccsA*, *ndhF*	*rps16*, *atpH*, *rps4*, *ndhC*, *accD*, *petA*, *psaJ*, *rpl33*, *rpl20*, *clpP*, *petB*, *rps15*
Landraces	*atpH*, *rps4*, *ndhC*, *psbJ*, *rpl33*, *rpoA*, *rpl32*, *ndhF*, *rpl2*	*rps16*, ***atpA***, ***atpF***, *atpH*, *atpI*, ***psbC***, *accD*, *cemA*, *psbJ*, *psaJ*, *rps19*, *ycf1*, *ndhF*, *rpl2*	*rps16*, *atpH*, *ndhC*, *accD*, *petA*, ***psbL***, *rpl33*, *rpl20*, *clpP*, *petB*
Breeding lines	*rps16*, *rbcL*, *rpl33*, *clpP*	*rps16*, *atpH*, *rpoB*, *psaA*, *rps4*, *ndhK*, *atpB*, *psaI*, *rps18*, *rpl20*, *petD*, *rpl36*, ***rpl16***, *ycf1*, ***ndhG***, *ndhF*	*rps16*, *petA*, *petB*, *rps15*

Table S3. N_{ST} values within cotton wild-to-domesticated complex.

	Wild	Wild-TI	Landraces	Landraces-TI	Breeding lines
Wild	0	0.050035	0.04922	0.60376	0.59823
Wild-TI		0	0.39994	0.97521	0.96575
Landraces			0	0.67161	0.66746
Landraces-TI				0	0.25000
Breedin lines					0

Figure S1. Region order comparison of chloroplast genomes of wild, landraces, and breeding lines of cotton. Each color signals a locally collinear block generated using the alignment in Mauve. A: the structure is highly conserved in all genomes. B: lime block (148,100-148,498 pb) is characteristic of wild genomes. C: green and blue blocks correspond to regions shared by domesticated genomes. D: mint green block is unique in landraces of Guerrero and the green variety of Oaxaca.

Figure S2. Tajima's *D* test of the overall chloroplast genome in 1kb windows. Tajima's D=0: neutrality; Tajima's D<0: alleles in low frequency, recent selective sweeps, recovery of variability. Tajima's D>0: alleles with intermediate frequency, severe phenotypes are eliminated, and the most common phenotype is preserved.

Figure S3. Phylogenetic tree of *Gossypium* inferred using RAxML from SSC dataset. Colored rectangles frame the genomic clades and shading color block marked the wild-to-domesticated cotton complex, pink shows the BL, Hutchinson's landraces and TI-landraces clade, wild clade in turquoise, landraces clade in green, and wild+landraces in purple. There are two main clades with representatives of nine genomic types influenced by the orientation and diversity of the SSC region.

Figure S4. Modification of genetic diversity (color circles) of plastome by introgression. W: wild; L: landraces; BL: breeding lines; GM: genetically modified varieties.

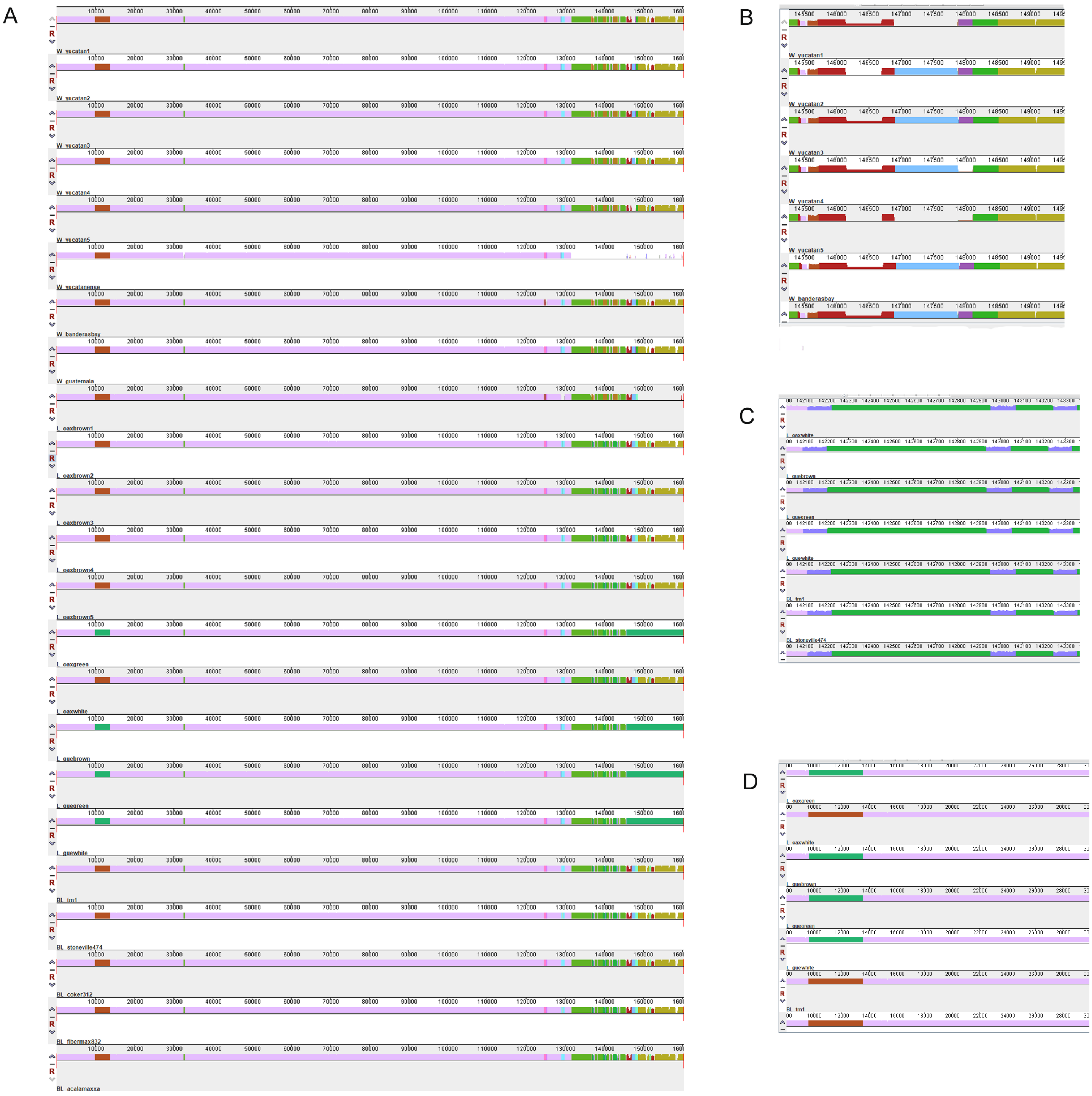
A
W_yucatan1
W_yucatan2
W_yucatan3
W_yucatan4
W_yucatan5
W_yucatanense
W_banderasbay
W_guatemala
L_oaxbrown1
L_oaxbrown2
L_oaxbrown3
L_oaxbrown4
L_oaxbrown5
L_oaxgreen
L_oaxwhite
L_quebrown
L_quegreen
L_quewhite
BL_tm1
BL_stoneville474
BL_coker312
BL_fibermax832
BL_acalamaxxa
B
W_yucatan1
W_yucatan2
W_yucatan3
W_yucatan4
W_yucatan5
W_banderasbay
C
L_oaxwhite
L_quebrown
L_quegreen
L_quewhite
BL_tm1
BL_stoneville474
D
L_oaxgreen
L_oaxwhite
L_quebrown
L_quegreen
L_quewhite
BL_tm1

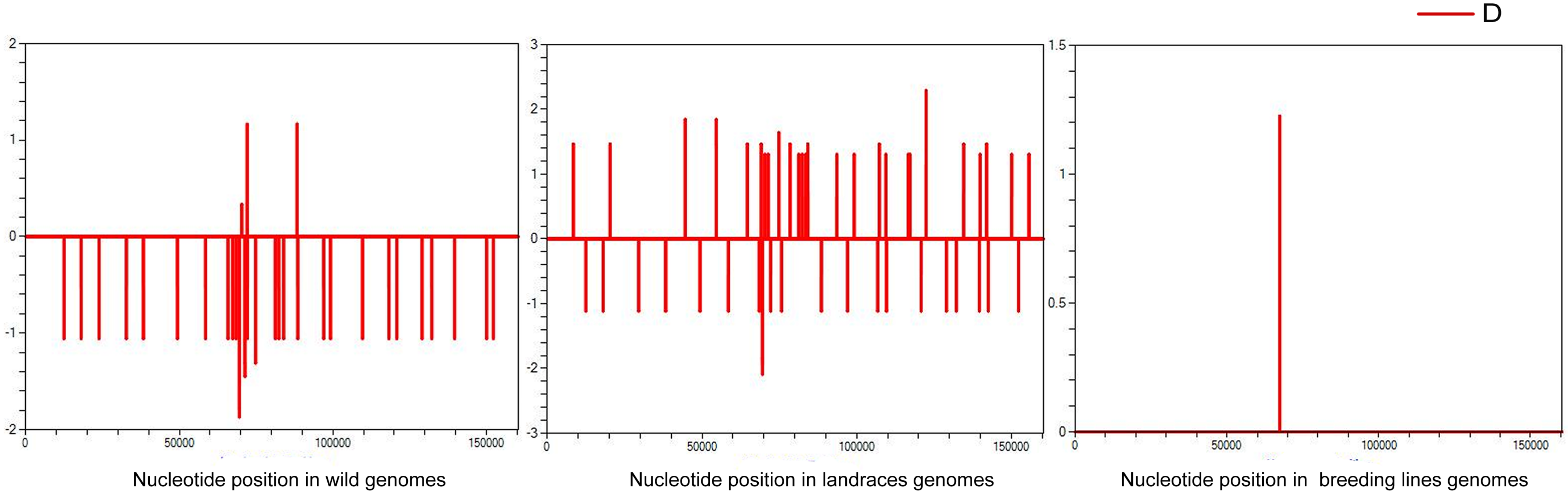
D
2
1
0
-1
-2
0
50000
100000
150000
Nucleotide position in wild genomes
3
2
1
0
-1
-2
-3
0
50000
100000
150000
Nucleotide position in landraces genomes
1.5
1
0.5
0
0
50000
100000
150000
Nucleotide position in breeding lines genomes

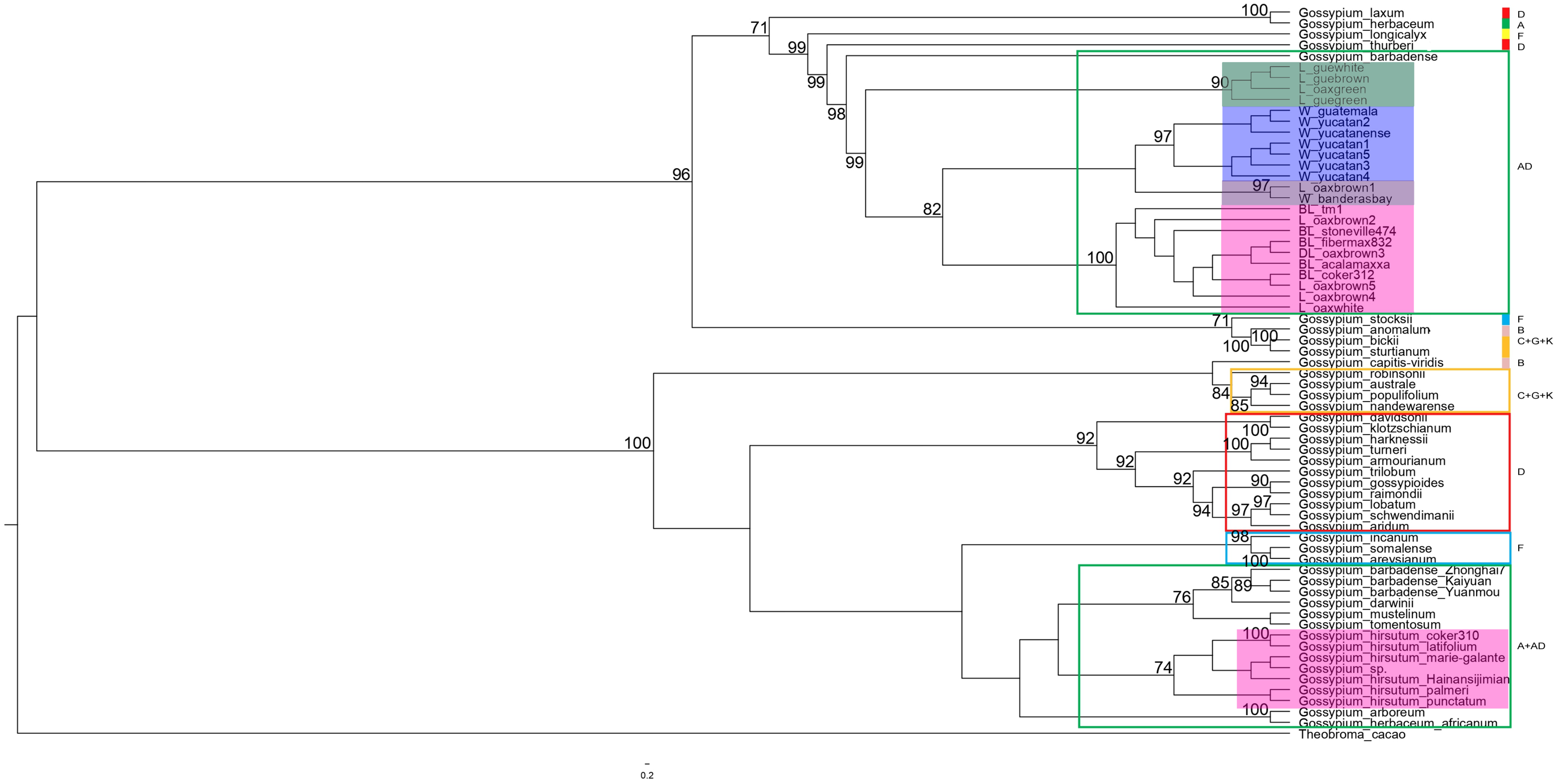
Gossypium_laxum
Gossypium_herbaceum
Gossypium_longicalyx
Gossypium_thurberi
Gossypium_barbadense
L_guewhite
L_guebrown
L_oaxgreen
L_guegreen
W_guatemala
W_yucatan2
W_yucatanense
W_yucatan1
W_yucatan5
W_yucatan3
W_yucatan4
L_oaxbrown1
W_banderasbay
BL_tm1
L_oaxbrown2
BL_stoneville474
BL_fibermax832
DL_oaxbrown3
BL_acalamaxxa
BL_coker312
L_oaxbrown5
L_oaxbrown4
L_oaxwhite
Gossypium_stocksii
Gossypium_anomalum
Gossypium_bickii
Gossypium_sturtianum
Gossypium_capitis-viridis
Gossypium_robinsonii
Gossypium_australe
Gossypium_populifolium
Gossypium_nandewarense
Gossypium_davidsonii
Gossypium_klotzschianum
Gossypium_harknessii
Gossypium_turneri
Gossypium_armourianum
Gossypium_trilobum
Gossypium_gossypioides
Gossypium_raimondii
Gossypium_lobatum
Gossypium_schwendimanii
Gossypium_aridum
Gossypium_incanum
Gossypium_somalense
Gossypium_areysianum
Gossypium_barbadense_Zhonghai7
Gossypium_barbadense_Kaiyuan
Gossypium_barbadense_Yuanmou
Gossypium_darwinii
Gossypium_mustelinum
Gossypium_tomentosum
Gossypium_hirsutum_coker310
Gossypium_hirsutum_latifolium
Gossypium_hirsutum_marie-galante
Gossypium_sp.
Gossypium_hirsutum_Hainansijimian
Gossypium_hirsutum_palmeri
Gossypium_hirsutum_punctatum
Gossypium_arboreum
Gossypium_herbaceum_africanum
Theobroma_cacao
D
A
F
D
AD
F
B
C+G+K
B
C+G+K
D
F
A+AD
0.2

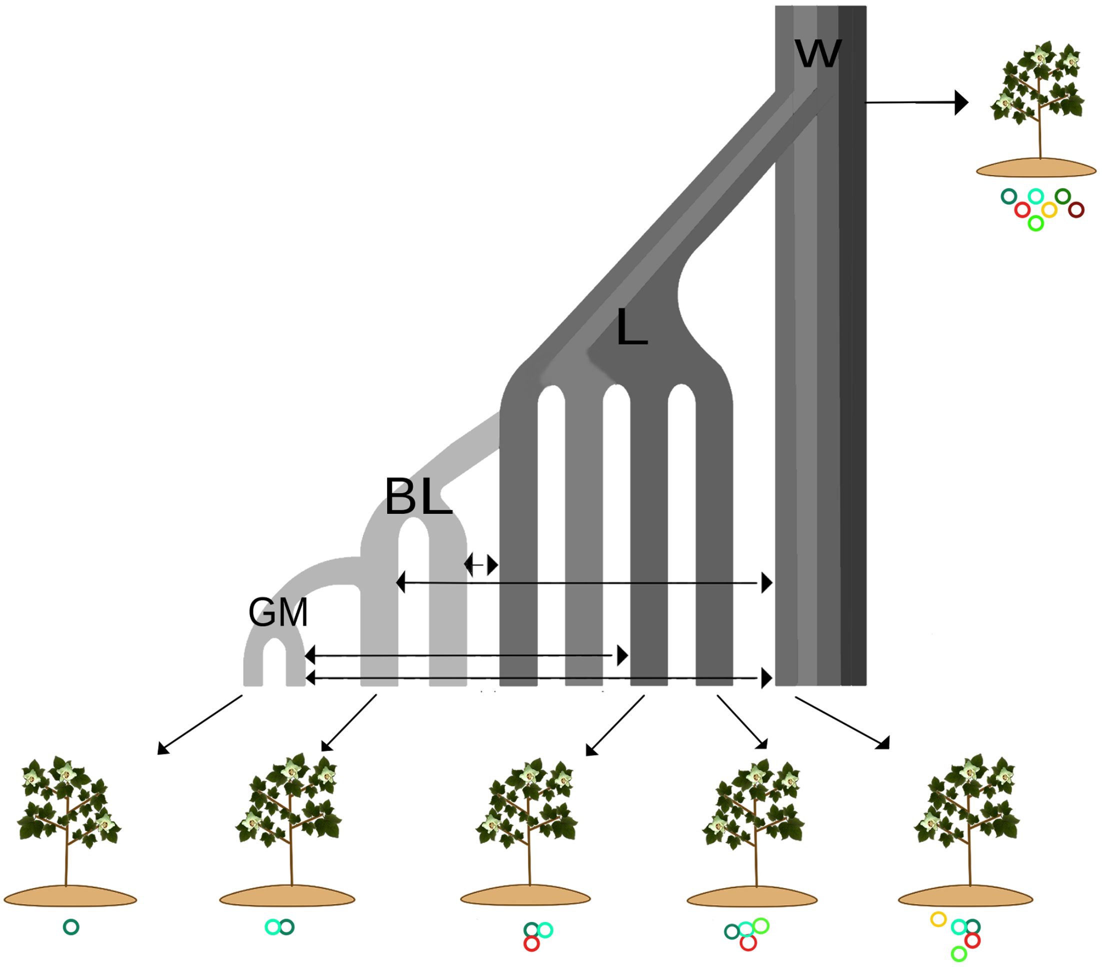
W
L
BL
GM
